# Book Review: Engaging Language Learners in Contemporary Classrooms

**DOI:** 10.3389/fpsyg.2021.726733

**Published:** 2021-08-05

**Authors:** Quanyue Wang

**Affiliations:** School of Chinese Culture and Communication, Beijing International Studies University, Beijing, China

**Keywords:** book review, language learning, contemporary classroom, social perspective, engaging learning

The main purpose of *Engaging Language Learners in Contemporary Classrooms*, written by two prominent applied linguists, is to fully understand how best to initiate and sustain learner engagement in contemporary language classes by embarking on a set of principles and teacher actions. It is documented that engagement is a multifaceted phenomenon, so its complexity and dynamicity have been well-represented and captured in this book by looking at leaner engagement from cognitive, emotional, behavioral, and social perspectives.

This volume consists of Forward, Introduction, and six chapters. In the introduction, the authors conceptualize student engagement in EFL/ESL classes, elaborate on the advantages of concentering on engagement in contemporary L2 classes, and justify why student engagement is essential. Furthermore, they postulate that engagement encompasses active involvement that is suitable for contemporary classrooms. The chapter also delineates the outline of the subsequent chapters.

The focus of Chapter 1 is to discuss the numerous contextual factors that can affect student engagement in L2 classrooms. The authors argue that sociocultural, educational, emotional, and linguistic factors impact student engagement; consequently, they foreground five maxims that enhance student involvement. These principles are as follows: (1) each language has a sociocultural status; (2) language learning needs to be connected to the authentic life beyond the classroom; (3) families can make a rich resource for student engagement; (4) “school priorities, curricular relevance and testing policies have a bearing on engagement” (p. 34); and (5) “whole-school culture can cultivate or kill learner engagement” (p. 37).

Chapters 2, 3, and 4 specifically focus on interpersonal and intrapersonal constituents that make the three cornerstones that are viewed to boost student engagement: “*the learner's psychological state*, their *relationship to the teacher* and their *relationship with their peers*” (italics is original, p. 45). For instance, Chapter 2 concentrates on learners' mindsets, beliefs, and feelings. The authors aptly enumerate the five facets of creating the learners' mindsets: “a sense of competence, a growth mindset, a sense of ownership and control over the learning process, confidence/willingness to be proactive and, finally, grit” (p. 70). Furthermore, the chapter also suggests five action strategies to facilitate these maxims “thinking and acting like a coach, making the learning progress visible, discussing beliefs explicitly, building choice and learner voice into the learning process, teaching learners how to learn” (p. 71).

Chapter 3 deals with one of the teacher-student interpersonal variables–teacher-student rapport. The authors stipulate that to boost learner involvement with teachers in relational terms, they are required to connect with them socially, affectively, cognitively, and behaviorally. The authors suggest six principles such as “being approachable, empathetic, and responsive to learner individuality, believing in all of your learners' potential to improve, seeking to support learner autonomy, and remaining passionate about what you do” (p. 95). They also round off the chapter by suggesting five teacher actions.

In Chapter 4, the authors argue that teachers play indispensable role in fostering positive peer relationships and the creation of peer values. They highlight the role of group dynamics in involving students, with an emphasis on peer relationships and classroom culture. This chapter brilliantly captures some principles and actions for teachers. The principles include “creating a safe environment for the learner group to develop and gradually become a mature, productive unit, characterized by cohesiveness and collaboration” (p. 130). The actions that teachers need to embark on to enhance positive group interactions for engagement encompass “mixing up learners, developing a sense of ‘we’ in the class, preparing learners for groupwork through building relevant interpersonal, collaborative and linguistic skills, structuring classroom life around the 3 ‘R’s—Rules, Roles and Routines, and fostering democratic participation” (p. 130).

Chapter 5 presents five principles and five teacher actions to initiate learner engagement on tasks. Thinking concretely about the actual learners, galvanizing students emotionally, creating curiosity, focusing on task set-up, and keeping learners active. The teacher actions to put into practice these maxims encompass commencing task engagement with purposefully small steps, provoking surprising factors, creating puzzles, designing cliffhangers, and embarking on questions to trigger curiosity.

The purpose of Chapter 6 is to sustain learner involvement in tasks. The authors highlight that initiating learner involvement is not enough, and we need to sustain their engagement. To do so, they suggest that teachers need to cater for cognitive challenge, maximize enjoyment, grab attention, employ the power of unpredictability, and acknowledge achievements. Using the power of stories, making the students the heroes of their tasks, giving rewards, breaking the tasks into smaller parts, and working with the principles are the five teacher actions which can sustain leaners' involvement. The authors close their mission by highlighting three major themes in learner engagement: “*the power of positive emotions, empowering learners as partners in their education* and *active participation”* (italics is original, p. 207).

This book is highly insightful and thought-provocative because not only does it provide robust theoretical rationales for each chapter, but also it offers down-to-earth principles and viable teacher actions that enable teachers to enact the maxims. Moreover, the book is replete with quotes and reflection tasks that can certainly entice and engage the readers throughout the monograph. What I specifically enjoy reading throughout the book is that the suggested teacher actions do not seem prescriptive, but rather they help to cognitively, emotionally, behaviorally, and socially engage learners and sustain their involvement. However, it is suggested that in the next editions, the authors can add rhetorical/relational goal theory (Mottet et al., 2006) to provide consolidated rationales for teacher-student interpersonal relationships. All in all, this book provides treasure trove of information for language students, teachers, teacher educators, and researchers who are interested to maximize learners' language learning and engagement.

## Author Contributions

The author confirms being the sole contributor of this work and has approved it for publication.

## Conflict of Interest

The author declares that the research was conducted in the absence of any commercial or financial relationships that could be construed as a potential conflict of interest.

## Publisher's Note

All claims expressed in this article are solely those of the authors and do not necessarily represent those of their affiliated organizations, or those of the publisher, the editors and the reviewers. Any product that may be evaluated in this article, or claim that may be made by its manufacturer, is not guaranteed or endorsed by the publisher.
